# Novice Nurses’ Experiences Caring for Acutely Ill Patients during a Pandemic

**DOI:** 10.3390/nursrep11020037

**Published:** 2021-05-27

**Authors:** Heather Naylor, Cynthia Hadenfeldt, Patricia Timmons

**Affiliations:** 1 College of Nursing, Creighton University, Phoenix, AZ 85012, USA; patriciatimmons@creighton.edu; 2 Sinclair School of Nursing, University of Missouri, Columbia, MO 65201, USA; 3 College of Nursing, Creighton University, Omaha, NE 68178, USA; cynthiahadenfeldt@creighton.edu

**Keywords:** COVID-19, nursing, pandemic, qualitative research, workforce

## Abstract

The Coronavirus pandemic erupted in 2020 and new graduate registered nurses (RNs) found themselves caring for those with devastating illness as they were transitioning into nursing practice. The purpose of this study was to describe the experience of novice nurses working in acute care settings during a pandemic. This qualitative phenomenological study of novice nurses working in facilities providing acute care for COVID-19 patients was conducted in Phoenix, Arizona, USA. Purposive sampling identified 13 participants for interviews. Data were analyzed using thematic analysis. Eight themes emerged: Dealing with death, Which personal protective equipment (PPE) will keep us safe?, Caring for high acuity patients with limited training, Difficulties working short-staffed, Everything is not okay, Support from the healthcare team, Nursing school preparation for a pandemic, I would still choose nursing. Novice nurses felt challenged by the experience and were at times overwhelmed and struggling to cope. Support from peers and coping skills learned during nursing school helped them continue to work during a critical time. Data from this study suggest that some participants may have been experiencing symptoms of anxiety, depression, or post-traumatic stress disorder, and findings provide foundational insights for nursing education and psychological interventions to support the nursing workforce.

## 1. Introduction

New graduate registered nurses (RNs) face stressful challenges learning their role as novice nurses. New nurses entering into clinical practice can face daunting issues: complex environments with advanced medical technology, high patient-to-nurse ratios requiring high-level skills, the need to advocate for patients and their families while delivering safe, quality care, and extending respect and compassion to individuals and their families. New graduate nurses may struggle with critical thinking skills, patient needs identification, and prioritization of patient care needs [1].

Without appropriate support, many new graduate RNs may experience stress, fatigue, anxiety, and burnout [2,3,4,5] and may leave the profession due to difficulty adapting to their role [6]. The stress of role transition accounts for up to 30% attrition of new graduate nurses in their first year [6], and up to 57% in their second year [7].

Undoubtedly, the well-documented stress of role transition is compounded when met with the severe strain of a global pandemic, and as cases of illness rise, the need for a robust workforce becomes paramount. Emerging evidence suggests that nurses have increased risk of stress, depression and burnout during COVID-19, with younger, less experienced female nurses at increased risk for mental health issues [8]. Providing appropriate support and resources during this transition is imperative to the satisfaction and retention of new graduate nurses; however, doing so is highly dependent on understanding the experiences of these key personnel during times of extreme stress. A recent meta-analysis found that a barrier to implementing appropriate interventions is lack of understanding of what staff and organizations need during pandemics to support their mental health [9].

While prior studies document Severe Acute Respiratory Syndrome (SARS) pandemic effects on nursing students [10], and the psychological impact on experienced nurses caring for COVID-19 patients in China [11], the current study captures the experience of novice nurses managing the stress of their professional role transition in a variety of settings during the COVID-19 pandemic which has not been previously reported. Heung and colleagues [10] suggested that working during a pandemic reinforced a strong sense of professional identity for nursing students in Hong Kong, China, during the peak of SARS. The study by Sun and associates [11] found that nurses with a median of 3.5 years of work experience similarly adapted to negative and positive emotions as well as personal growth in response to caring for patients with COVID-19. The purpose of this study was to describe the experience of novice nurses with less than two years experience working in acute care settings during a pandemic.

## 2. Materials and Methods

Phenomenology is a philosophical approach and qualitative research method that can be particularly effective when studying phenomenon where little knowledge has been previously uncovered as in the phenomenon experienced by nurses caring for acutely ill patients during a pandemic. Phenomenology is the study of an individual’s lived experiences and helps to develop a better understanding of what the experience means [12,13]. The purpose of the research method is to describe the reality of an experience and the effect that the experience has on the person [12,13]. Phenomenology has a unique approach to data collection in that the researcher is the primary study instrument and the subject’s story is the data [12,13]. Data collected through phenomenological methods may be the basis for the development of quantitative measures utilized in future studies. Researchers must bracket their own perceptions and views during data collection and analysis so as not to bias results [12].

### 2.1. COREQ Checklist

Methodological data were presented in this section using the consolidated criteria for reporting qualitative research (COREQ) checklist for qualitative research to ensure explicit and comprehensive reporting. The COREQ checklist is a 32-item checklist for interviews to ensure rigor in reporting in qualitative studies [14].

### 2.2. Participants

Once university institutional review board approval was received, recruitment of novice nurses was conducted in Phoenix, Arizona, a large southwestern city and the fifth-most populous city in the United States. Purposive sampling was employed to recruit novice nurses with invitations via email listserv of one university’s accelerated nursing program alumni, and social media posts on a page maintained by those nursing alumni. Participants were invited to respond with their interest via email, call, or text. Participants were recruited to represent a broad sampling of specialties (e.g., medical/surgical intensive care, labor and delivery, inpatient psychiatry). Care was also taken to include participants working in various acute care settings throughout the city with or without direct contact with COVID patients as part of their daily work assignment.

Participants were novice nurses with two years or less experience working full-time in any acute care setting that cared for patients with COVID-19. Six different acute care facilities were represented. Ten nurses worked with COVID patients or patients under investigation (PUIs), and three worked on non-COVID units, with one of these three asked to cross-train to work in a COVID unit. Nurses were graduates of Creighton University College of Nursing’s one-year accelerated program who had already completed another Baccalaureate Degree in an unrelated field. Consequently, students were older than traditional nursing students. The program is based in Phoenix, Arizona, and participants graduated with Bachelor of Nursing Science degrees. Specialties represented in the sample included: neurological intensive care unit-turned-COVID unit, emergency, telemetry, medical/surgical, labor and delivery, adolescent psychiatry, neonatal intensive care, trauma step-down, medical oncology, observation, and float pool. The sample included 13 nurses, of which three were male and 10 were female. Two additional participants expressed interest but did not respond to a follow-up inquiry to schedule an interview. All who responded and scheduled were interviewed. The average age of participants was 29 years with a range of 24–41 years. The average time since graduation from nursing school was 13 months, with an average time working of 11 months (range: 6–18 months) (see Table 1).

### 2.3. Interview Procedures

All interviews were conducted by the principal investigator (PI) who is a PhD student at the University of Missouri and an Instructor in the College of Nursing at Creighton University. The PI is also employed as a nurse practitioner on the acute pain service at a large tertiary care facility in Phoenix, AZ. All participants were former students of the PI and one of the co-authors. Though the PI was working at the hospital during the pandemic, she did not provide regular or significant direct patient care to COVID patients. Care was taken to bracket her personal experiences during data collection to minimize bias. The PI shared with participants that she was interested in capturing their unique and important perspective of working during a pandemic as a new nurse with intent to share findings broadly through scholarly presentation and publication because little research exists to describe this experience.

Individual, private interviews were conducted with face-to-face video teleconference technology to allow for appropriate social distancing. Each interview lasted approximately one hour, extending beyond this timeframe at the discretion of the participant. No repeat interviews were conducted. Interviews were recorded via the teleconference technology along with a digital audio voice recorder with participants’ permission. A recording feature of the teleconferencing technology was used to capture verbatim dialogue of interviews. Transcriptions were cross-referenced with the digital recordings for accuracy by the PI. Written field notes were also collected by the PI during the interviews.

A semi-structured interview guide was used to collect demographic information and responses to open-ended questions (see Table 2). Broad questions were asked, such as, “What are your thoughts about working during this pandemic?” and “Has it affected you personally, and if so, how?” Probing follow-up questions were used as appropriate such as, “Tell me more about that.” One participant emailed a one-page blog post she authored in advance of her interview that detailed her experience, and offered her permission to include this as an artifact with her interview transcript for analysis. After interviewing 13 participants, no new information emerged, and it was determined that saturation had been reached and data collection was complete. As two methods of recording interviews were used and cross-referenced for accuracy by the PI, transcripts were not returned to participants for correction.

### 2.4. Data Analysis

For the data analysis, a team of three researchers, including the PI, independently examined and coded the 13 de-identified transcripts using Microsoft Word and “track changes” and “comments” features with notes on sidebar. Themes were derived from the data. Transcript data were analyzed using qualitative thematic analysis as described by Castleberry and Nolen [15]. In this approach, data are comprehensively analyzed through a systematic five-step process: compiling, disassembling, reassembling, interpreting, and concluding. Compiling involves transcribing data into a usable form for analysis. Transcription and data organization were completed by the PI, which allowed for an intimate knowledge of transcription data. Disassembling is a process of parsing data and searching for meaningful connections through coding and identification of similarities and differences. This was accomplished through color-coding and highlighting by reviewers. In reassembling, data are analyzed in context with each other, and themes and subthemes begin to emerge. The interpretation phase, which can and should occur throughout the process, involves the discussion of relationships between themes supported by raw data or quotations from transcriptions. Data were analyzed through independent review of transcripts by researchers, then bringing themes to the group for discussion. If a difference in interpretation of the themes arose, the researchers revisited the transcripts and negotiated a common understanding. Concluding is the final phase during which research questions are answered based on the previous data analysis [15]. Member checking was performed by sending the results to the participants, and accuracy of the results was confirmed. Due to the smaller sample size, budget, and time constraints, no qualitative data analysis software was used to manage the data.

## 3. Results

Participants described challenges of dealing with frequency of patient deaths, high-acuity patients, changing personal protective equipment (PPE) requirements, and working short-staffed. They also described positive aspects such as bonding with teammates, satisfaction with their decision to become a nurse, and how nursing school featured in their adaptation to working in a pandemic. Below is a summary of these findings with supporting quotes. All participants were assigned pseudonyms to protect their privacy.

### 3.1. Dealing with Death

Of concern to many participants were the difficulties with increased frequency of experiencing the death of patients despite intense efforts to keep them alive. They felt responsible to be there for their patients during end of life when family were not able to be there. Gabby described, “So most of my patients with COVID die. So if they’re in the ICU, they don’t leave the ICU alive. So, I think that’s a big fear, just knowing I’m the last person to be there with them because most of they don’t make it.” Participants described being greatly impacted by each passing, but after a while, could not recall each patient in their care who had died. Irena put it this way: “The amount of loss that I’ve seen since March, at first it was always in front of my mind. I would say, I’ve lost four patients so far, I’ve lost six patients so far and now the sad truth is, I can’t even keep track anymore.”

Emma noted that even her experienced nurse colleagues had never experienced so many deaths, “A lot of the nurses that I worked with have literally never experienced a death, even in five or six years of nursing. Now we’re seeing it every shift so it’s a big adjustment for us. We’re used to having patients have good outcomes.” She also noted that the Ethics Committee was needed more frequently to make final, end-of-life decisions for patients. Amy noted the emotional toll of withdrawing care: “When you have to sit there and withdraw care at the bedside, it kind of just takes a little bit of your soul away every time you have to do it.”

### 3.2. Which PPE Will Keep Us Safe?

Participants expressed concerns about how to protect themselves in patient rooms, and how the guidelines for personal protective equipment (PPE) changed frequently in the early stages of the pandemic. They sometimes experienced daily changes to PPE protocols. Jane and Hattie shared their perspectives on PPE requirements they described as changing each shift:

Jane noted, “Things were changing every day with their recommendations saying you can wear two masks and then no, it’s not a good idea to wear two masks and you can use and reuse these certain types of N95s and things were just changing all the time and it kind of felt like we weren’t ever getting the most accurate information which is pretty scary…so every shift, things were changing and the protocols were different. So it just kind of felt like chaos.”

Hattie said, “I think they’re trying their hardest to keep us up to date on the rules, but one day we’re wearing safety glasses. The next day, you only wear them if you’re in rule-out rooms…later everyone would be going to the COVID unit if they’re rule-outs and then for a week, rule-outs would come to the floor.”

Nurses felt some PPE decisions were made based upon available supplies. Jane said, “It was hard to know if changing PPE requirements were based on new evidence and knowledge or because the hospital has to mitigate low stock of N95s.” They worried about personally getting COVID and taking it home to family members. Irena put it this way

“My first reaction I had the first day that I heard that I was going to a COVID unit was sheer fear…so much unknown about the virus and how it spreads and how we protect ourselves. They would allow us to go into the patient rooms with just surgical masks on. And we weren’t wearing N95s as long as we stood within this box that they had taped off on the floor, because if you were 6 feet away from the patient, they thought you were safe.”

### 3.3. Caring for High-Acuity Patients with Limited Training

Participants reported that the medical demands of COVID patients required them to pivot quickly to caring for patients of a much higher acuity. As Emma stated, “the level of acuity of my patients increased exponentially overnight with no training and we just had to go with the flow and figure out how to handle it.” Many felt inadequately prepared for this transition, as Irena described, “The reality for us is that we basically had an ICU situation on almost every floor that we were on, so our house supervisor basically said, you’re working on an ICU unit without ICU training right now.” Nurses mentioned that managing critically ill patients is stressful for new nurses under normal circumstances, but the pandemic compounded the stress. Bill gave an example of caring for a challenging trauma patient with multi-organ system dysfunction who also had COVID, “they’re not only a trauma patient but now they’ve contracted COVID, so where they would otherwise not need aggressive respiratory support, now they’re needing respiratory support.”

Nurses described an increase in daily code events and uncertainty about code outcomes that added to their stress. Denise noted a particularly difficult two-week time period during the pandemic’s peak, “The ER (emergency room) is one of those units where it does happen from time to time…I’ve never had so many codes in one short time frame.” Many described uncertainty about whether they would recognize key symptoms that indicate clinical decline. As Irena stated, “It’s just always in the back of your mind. Am I doing enough? Did I catch everything? You know, did I miss something…I think you’re always kind of second-guessing yourself, especially as a new grad because you’re still learning what all that stuff sounds like and feels like and presents as.” Participants also described uncertainty with the new disease process of COVID itself, and the speed at which symptoms appear and patients deteriorate. As Marie said, “The other thing with COVID is just, it just happens so quickly. These patients are declining so rapidly.”

### 3.4. Difficulties Working Short-Staffed

Participants described problems with staffing. Many mentioned staff quit their employment, which participants attributed to fear of working during the pandemic. As Fernando put it,

“You have people that are pregnant, you have people that have small children, you know, everybody has families. Everybody wants to keep safe and so you almost immediately had tons of staffing problems. And you have people calling out because they’re scared.”

Some of the participants described nurses who quit to take more lucrative travel nursing jobs. Others described colleagues who became burned out and quit. Staffing shortages were not limited to nursing but also patient care technicians and certified nursing assistants, adding to nursing workloads. As Marie noted, “We were kind of forced to take on the role of a tech and a nurse with higher acuity patients. So, I think day in and day out for a few weeks there it was very stressful for those reasons.” Because of the shortage, nurse-to-patient ratios increased as did overtime requests. Participants worked extra shifts for a variety of reasons, such as wanting to help the team. Bill described picking up extra hours as, “It’s exhausting. But I like what it stands for and being able to help and be there for the rest of my team and the patients.” Others worked extra, sometimes excessively, for financial incentives. Emma said, “There used to be all sorts of rules and regulations in place of how many shifts in a row we can work and how many shifts in a pay period you can pick up, but desperate times call for desperate measures, and there were no rules. Some people just did crazy things to make crazy amounts of money.”

### 3.5. Everything Is Not Okay

Many individuals described the difficulties in caring for very ill patients, the loss of life, and how it was impacting the nurses’ ability to cope. Nurses reported nightmares and difficulties with sleeping, depression, anxiety, and fear as they watched the suffering and took the emotional toll home with them. They expressed that they were not able to get away from COVID. As Emma noted,

“You leave work and you get in the car and the radio’s on and all anybody is talking about is COVID so then you have to turn that off. And then you call somebody to decompress while you’re driving home and all they want to talk about is COVID. And then you get home and you turn on the TV and it’s COVID.”

Participants also worried about their patients long after their shifts ended. Irena admitted, “I’m not doing okay. I’m like, I’m not sleeping. I’m having trouble eating. I’m worrying about my patients all the time. Like I drive home and I am just sobbing like the whole way home. And I just, I don’t know what to do.” Nurses expressed that the situations they were experiencing are hard to handle emotionally. Layla put it this way: “So I think it is incredibly depressing. Not only from the work aspect, but then trying to go home and pretend everything’s fine, it’s okay, when it’s not.” They had difficulty processing the events they had seen and moving on with their lives, and noted that their families were not able to fully understand what they were going through. As Amy described,

“Being a nurse is hard every day. There are people who die every day and you can’t come home and talk to your significant other and spill out all on them, because they didn’t sign up for that. That’s not fair. And you can’t come home and be negative…and it doesn’t feel like they even understand or comprehend or can really be there for you because they don’t.”

Nurses not working with COVID patients were not immune to the stress as they experienced guilt that their co-workers were overwhelmed and they were not there in the middle of it trying to help them. Karen summed it up: “I almost had some guilt that I know some of my classmates are working on the COVID units and they’re just stressed and overwhelmed and I just felt like I needed to be a part of that, too.” Irena shared with the authors a blog post that she wrote prior to her interview regarding her experience (see Box 1).

Box 1 **Participant Irena’s unsolicited blog post received via email in advance of interview.**

5 August 2020

 

“Hi,

 

I thought I would share one of my latest Blog entries with you about my experience with COVID. I share my thoughts and frustrations with a small group of friends and other RNs as I find it helps to be able to have a way to express feelings during this unprecedented time.

 

Irena (pseudonym)”

 

“The sweat is running down my back as I blink to try to see through my foggy, streaked face shield. The beeping of the machines just will not relent. My patient is dying, and I know it. I fumble with the phone clipped to my scrubs underneath my paper thin yellow gown, desperate to call for help. I am alone in this isolation room with my patient and COVID 19. The oxygen saturation monitor keeps dropping - 84, 80, 76. I call for help but no one comes right away. Our hospital is bursting at the seams with COVID patients and there is no one to help. After what seems like an eternity, someone enters the room in head to toe protective gear and we start the dance. We begin carefully untangling the lines, tubes, and wires connected to my patient, we need to get him lying on his belly as quickly as we can. The moving is exhausting. My body aches and my patient is in excruciating pain from just turning over because this virus is relentless and unforgiving. I have been doing this for 127 days now. The sweating, the praying, the dancing, and the grieving. My patient is moaning on his belly now, his oxygen is still not improving despite giving him all the oxygen we can. I stroke his arm and tell him to take some deep breaths and that we are going to help him. I know I am lying because I have seen this too many times to count. I make the call to the ICU and when a bed opens up (after someone expires) they will whisk him away to be intubated. I just hope I can keep him alive that long. My other 4 patients are waiting in their own rooms of isolation hell and they are all very sick. They are all on oxygen, COVID positive, struggling to breath, desperate, and all alone. I make the phone call to my patient’s family and tell them that their loved one is not improving, and the next step will be intubation. I hear the all too familiar sobbing and begging me to let them speak to the patient, but the patient can’t speak through the tight mask blowing life into his lungs.

 

How did I end up here? A new graduate RN in the middle of a global pandemic. I can only describe it as being on a long, grueling hike up a mountain to finish nursing school and pass the NCLEX exam and when I finally make it to the top, I am thrown off the edge of the cliff. No parachute, no training, no lifeline. Here I am at 41 years old finally working at my dream job of being a Registered Nurse and I am in hell.”

### 3.6. Support from the Health Care Team

Participants talked about the importance of having support from other nurses and health care workers who understood what they were experiencing and the demands of caring for very ill, often terminal, patients. Nurses described strategies that had emerged to provide support such as texting and calling each other throughout the day and after their shift to offer support, establishing a group email to discuss feelings about difficult patient situations, and meeting up for lunch at work. Irena described how the group email strategy helped her cope:

“On difficult days we would “reply all” to a group email at work and start an open dialogue where there was no judgment…We would read it and know that we weren’t alone in it. Even if it was just a quick email like ‘I feel like I’m drowning—I don’t even want to go back to work tomorrow it’s so bad’. People responded and said ‘Hey, I’m working tomorrow, let’s meet up for lunch. Let me know when you’re taking your break’. Just knowing that you have that kind of support, especially as a new nurse and somebody who’s brand new at the hospital…this kind of helped to bring me into a little circle which felt good to be able to get out feelings and frustrations in a no judgment zone.”

Family and friends attempted to be supportive but the nurses did not feel that they understood what the nurse had just experienced. Marie commented, “The support at the hospital has been incredible. The way the team and all the nurses have just really been doing an amazing job of coming together and supporting one another, whether it’s going into a room to help with a task or it’s some mental support at the end of the day. The last couple months were really tough and I think about how much we all have helped one another.”

### 3.7. Nursing School Preparation for a Pandemic

Each participant expressed both satisfaction and limitations in their nursing education, describing the coursework as sufficient but the application to practice specific to pandemic was missing. Many described feeling unprepared and, at times, powerless as they were required to adhere to standards of care changing rapidly and practices, like reusing PPE and increasing nurse to patient ratios, became commonplace. They recalled learning about pandemics, viruses, and emergency preparedness during their educational program; however, participants were ill equipped to employ related nursing protocols in the context of a large-scale pandemic. This gap was not seen as detrimental to their success because working as a nurse during a pandemic was unknown to all nurses they encountered. Emma describes this imparity:

“I don’t think there’s anything that could have fully prepared us for the pandemic. I mean, we learned about pandemics. We learned about viruses, you know, we learned all these things, but I don’t think there’s anything that can prepare you for just being thrown into it.”

Marie praised her program for imparting needed information but was aware of the incongruence between didactic education and bedside practice: “I think the reality of nursing school is that not everything is going to copy over directly onto the job, but I think my, I think our school did a good job of doing their best.” Cesar noted his colleagues who were experienced nurses reverted to novice nurses when faced with the uncertain challenges of working during a pandemic. He felt that this was evidence that nursing programs cannot fully prepare you:

“…The veteran nurses on the floor and they’re like, yeah, we’ve kind of seen nothing like this either and they’re the people you kind of go to, to ask questions. So, if they didn’t know what was going on, and I mean, I don’t think there’s anything that could have made me more prepared.”

Conversely, many believed that aspects of their academic program effectively prepared them to assume the role of the nurse. They noted that clinical experiences were valuable as were post-clinical debriefings, which allowed students to discuss clinical based topics among their peers. Marie believed that hands-on clinical were “the most beneficial” aspect of nursing school, specifically, post-clinical debriefing:

“I found that to be so helpful, whether it’s debriefing regarding a task of medication or whole patient experience that we had to deal with that day… just being able to sit down with a group, a smaller group of people and really just talk about things.”

Denise echoed this opinion that sharing experiences after a clinical was important: “I would say that definitely helped because even though I didn’t have like an experience, it was nice to hear like other people’s experience.”

Participants described the ability to manage time effectively, employ flexibility, and put into practice new skills was a result of learning in an accelerated nursing program. The pace of the twelve-month accelerated nursing program assisted them in developing critical thinking and prioritization, skills they utilize in their bedside practice. Emma states “Going through the accelerated program and having to learn to manage all of that and like keep myself on a schedule is really helpful with keeping my two patients on a schedule.” Marie compared her experience in an accelerated nursing program to working during a pandemic. She states “I think it prepared us pretty well especially because the program was only a year long. And so, we were forced to kind of put our heads down and grind for a year. And that is kind of what it feels like.”

### 3.8. I Would Still Choose Nursing

Many participants entered nursing as a second career. They voiced little to no negative feelings about their decision to become a nurse; many felt certain that they would still pursue nursing as a career, even if a global pandemic was in their future. Jane was glad she entered the field, “I love my job and what I do, especially the specialty I’m in.” Similarly, Hattie described a passion for nursing, “I absolutely love my job. There is not one aspect of it that I don’t like.”

There was overwhelming confidence in their role choice and belief that they make a difference in the lives of their patients. Bill stated: “I still would do it all over again. If I knew that this was at the end of the tunnel, I 100% would. I love what I do. I definitely feel that I am helping people each and every day at work.” Irena is similarly confident in her decision to become a nurse. She stated:

I absolutely would do it again, a million times. I love being a nurse. I love helping people. I actually have been nominated twice for Daisy awards at my hospital which is a huge big deal….I don’t regret my decision to become a nurse, especially as like a second career.

Though many participants were happy with their decision to be a nurse, many describe a desire to move away from bedside in the future. Several of the participants expressed a need to work in a different (non-inpatient) environment. Irena states: “I definitely have days where I’m like, I need to get out of the hospital. I need to get out of this COVID unit. I don’t know how many more times I can double glove and wear masks and, you know, do all this stuff. So always nursing, for sure, just maybe in a different environment.” The impact of death has affected Layla and her desire to stay in the inpatient setting. She stated, “I don’t think that I’ll stay like inpatient forever, or anything like that. I think, I think this experience helped me realize that for some of us, you know, outpatient where you’re not dealing with people dying left and right would be nice.” Marie expressed a desire to enter a field that focused on research: “... this has all made me want to go back to school, maybe for public health in the future…research and epidemiology is cool.”

## 4. Discussion

In this study, the experience of being a new nurse during a pandemic left nurses feeling that they were being challenged by the experience and that, at times, they were overwhelmed and struggled to cope with the intensity of the experience. Nurses found that patients were of high acuity and they felt they lacked adequate training to care for them, many patients did not survive despite the best medical and nursing efforts, guidance about PPE was regularly changing, and units were working short-staffed. In a study by Garcia-Martin et al. of novice nurses in the Emergency Department (ED), similar themes emerged from an analysis of 16 semi-structured interviews [16]. One of the themes identified was titled “Fears and concerns” which addressed the nurses caring for highly complex patients with limited training in the ED and fear of becoming ill or taking the illness home to family [16]. A subtheme called “Dealing with new challenges” under a separate theme spoke to the confusion surrounding appropriate PPE to be worn to remain safe and free of illness [16].

In this study, support from colleagues who understood the experiences of caring for these extremely ill patients helped nurses when the process of caring for patients had become difficult for them to handle. While the Garcia-Martin et al. article identified a theme of “Support for novice nurses,” the subthemes focused primarily on the need for information and organizing resources to provide care for the patients during a short stay in the ED setting [16].

One of the themes unique to this study was “Everything is not okay” which spoke to the personal coping of nurses who were caring for patients during the pandemic. Some participants described experiencing signs and symptoms such as inability to eat and sleep, nightmares, crying over difficult work situations, reluctance to return to work, and feeling depressed. that are potential evidence of extreme anxiety and stress, depression, or burnout and intention to leave nursing. In an article by Chen et al., the authors looked at predictors for leaving the nursing profession during the pandemic and concluded that clinical stress and frequency of caring for patients with COVID-19 infections impacted the nurses’ decision to stay in their career [17].

A systematic review of the literature by Carmassi et al. concludes that post-traumatic stress disorder (PTSD) was present during the first two pandemics and may be present in nurses caring for acutely ill patients during the recent pandemic [18]. An article by d’Ettore et al. also suggests that health care workers have experienced mental health issues during previous epidemics, such as SARS and Middle East respiratory syndrome (MERS) [19]. PTSD is a disorder characterized by recurring symptoms such as bad dreams, flashbacks, avoidance, hyperarousal, difficulty sleeping, and negative thoughts that develops in some people following exposure to a traumatic event [20]. In the Carmassi review, the authors identified exposure level to ill patients, years of work experience, female gender, and a number of other risk and resilience factors as determining if individuals might experience PTSD in the performance of their duties during a pandemic [18]. The International Council of Nurses published a COVID-19 Update suggesting that globally, nurses are experiencing burnout and mental distress associated with extreme pressures of working during the pandemic and number of nurse deaths exceeded 2200 as of January 2021 [21]. In this study, even those nurses without daily direct contact with COVID-19 patients experienced negative psychological effects, such as guilt that they were not doing enough to assist fellow co-workers.

Uncovering the experiences of novice nurses in a pandemic and understanding the unique challenges being faced provides a foundation for educational interventions that have the potential to strengthen and preserve the nursing workforce. In the theme “Nursing school preparation for a pandemic” students described how the demands of nursing school had instilled the ability to plan, organize, think critically, and be flexible in their practice. Clinical rotations and high intensity simulation experiences, post-clinical debriefings, and end-of-life or palliative care discussions were also helpful. In the article by Sparacino, role transition as novice nurses is inevitable but expert, caring, professional experiences with faculty can make a real difference for students in knowing how to engage in a positive way in their professional role especially during stressful times [6]. This education must continue to be enhanced with a focus on self-care and effective team communication.

For nurses in the stressful situation of providing care for patients in a pandemic, hospital management must provide additional support such as counseling sessions at no cost for nurses experiencing symptoms of anxiety, depression, or PTSD, nurse mentoring programs pairing nurses with several years of nursing experience with novice nurses, and providing structured weekly opportunities for debriefing with nursing unit managers, administrators, and colleagues to provide support. Staff educational sessions on the most effective techniques in caring for ill patients and preferred PPE equipment should be conducted.

Participants were asked whether they had used any resilience resources since the pandemic began. Interestingly, only one of 13 participants reported using employer-provided resilience resources, which included support webinars, counseling hotlines, and talking with hospital chaplains. Many sought their own forms of support with physical activity, media vacations, engaging in pleasurable and distracting hobbies, seeking help from their own counselors, or talking with friends and family members. This may point to a lack of understanding on the part of organizations about what resources are most needed by novice frontline nurses, and how best to connect employees to employer-offered resources, which supports findings of the meta-analysis by Pollock and colleagues [9].

### Limitations

Although the teacher-to-former student relationship of the PI to participants may be a limitation regarding perceptions about nursing school preparation for the pandemic, participants readily responded to calls for participation and shared openly with the PI, perhaps due in part to the established relationship. Additionally, participants noted in a theme that talking with other health care providers who understand was a significant and desirable form of coping with stress during this time, and they may have perceived benefit in sharing this experience with a fellow clinician, and their former teacher.

## 5. Conclusions

Understanding the experiences of novice nurses caring for acutely ill patients during a pandemic and how best to support these nurses was of keen interest to the researchers. The authors believe that this understanding was achieved in the context of this study. Implications of this study include further investigation of the signs and symptoms nurses experienced that were suggestive of anxiety, depression, and PTSD. These nurse participants should be studied longitudinally to uncover the impact on future nursing practice. While nursing education programs provided resources considered helpful for dealing with pandemic-related stressors, programs should build mechanisms into curriculum to focus on crisis management, post-clinical debriefing, strong team communication, and self-care. Employers should build time into the end of shifts for mandatory debriefing to support nurses’ mental health.

## Figures and Tables

**Table 1:** Participant Attributes (*n* = 13).

Variables	Categories	*n*	%
Gender	Female	10	76.9
	Male	3	23.1
Education	Baccalaureate Degree Nursing	13	100
	Baccalaureate Degree Non-Nursing	13	100
Age	24–29	9	69.2
	>30	4	30.8
Type of unit	COVID Intensive Care	3	23.1
	Emergency	1	7.7
	Medical/Surgical Telemetry	1	7.7
	Medical Oncology	1	7.7
	Trauma Step-Down	1	7.7
	Labor and Delivery	1	7.7
	Neonatal Intensive Care	1	7.7
	Adolescent Psychiatry	1	7.7
	Observation Telemetry	1	7.7
	Pediatric Float Pool	1	7.7
	Critical Care Float Pool	1	7.7
Daily exposure to COVID-19 or PUIs	Yes	10	76.9
	No	3	23.1
Amount of experience as nurse (years)	<1	9	69.2
	1–2	4	30.8
	>2	0	0

**Table 2:** Semi-Structured Interview Guide.

Demographic Questions	Open-Ended Questions
(a) Gender identity? (b) Age? (c) How long have you been out of nursing school? (d) How long have you been working as a nurse? (e) Do you work full-time, part-time, PRN? (f) What type of floor do you work on? (g) Have you used any resilience resources (counseling, mental health webinars, etc.) since the pandemic began? (h) Do you work with COVID-19 patients or PUIs as part of your daily job? (i) Have you been cross-trained or asked to float to COVID units?	(j) What are your thoughts about working during this pandemic? Has is affected you personally? How? (k) What motivates you to work extra shifts offered by your institution during the COVID crisis? (l) What fears do you have about working in a COVID environment? (m) How does your place of employment protect your health? (n) How well do you feel your academic program prepared you to work in this environment? What helped? What more could have been done? (o) What resources would be helpful to you right now? (p) How do you feel about your decision to become a nurse?

## Data Availability

The data presented in this study are available on request from the corresponding author. The data are not publicly available due to privacy protection.
